# Evaluation of the safety and immunomodulatory effects of sargramostim in a randomized, double-blind phase 1 clinical Parkinson’s disease trial

**DOI:** 10.1038/s41531-017-0013-5

**Published:** 2017-03-23

**Authors:** Howard E. Gendelman, Yuning Zhang, Pamela Santamaria, Katherine E. Olson, Charles R. Schutt, Danish Bhatti, Bhagya Laxmi Dyavar Shetty, Yaman Lu, Katherine A. Estes, David G. Standaert, Elizabeth Heinrichs-Graham, LuAnn Larson, Jane L. Meza, Matthew Follett, Erica Forsberg, Gary Siuzdak, Tony W. Wilson, Carolyn Peterson, R. Lee Mosley

**Affiliations:** 1 Department of Pharmacology and Experimental Neuroscience, University of Nebraska Medical Center, Omaha, NE USA; 2 Neurology Consultants of Nebraska, PC and Nebraska Medicine, Omaha, NE USA; 3 Department of Neurological Sciences, University of Nebraska Medical Center, Omaha, NE USA; 4 Department of Neurology, The University of Alabama at Birmingham, Birmingham, AL USA; 5 Great Plains Center for Clinical and Translational Research, University of Nebraska Medical Center, Omaha, NE USA; 6 Department of Biostatistics, University of Nebraska Medical Center, Omaha, NE USA; 7 Scripps Center for Metabolomics, Scripps Research Institute, La Jolla, CA USA; 8 Departments of Chemistry, Cell and Molecular Biology, and Integrative Structural and Computational Biology, Scripps Research Institute, La Jolla, CA USA

**Keywords:** Parkinson's disease, Neuroimmunology, Chronic inflammation, Immunotherapy

## Abstract

A potential therapeutic role for immune transformation in Parkinson’s disease evolves from more than a decade of animal investigations demonstrating regulatory T cell (Treg) nigrostriatal neuroprotection. To bridge these results to human disease, we conducted a randomized, placebo-controlled double-blind phase 1 trial with a well-studied immune modulator, sargramostim (granulocyte-macrophage colony-stimulating factor). We enrolled 17 age-matched non-Parkinsonian subjects as non-treated controls and 20 Parkinson’s disease patients. Both Parkinson’s disease patients and controls were monitored for 2 months for baseline profiling. Parkinson’s disease patients were then randomized into two equal groups to self-administer placebo (saline) or sargramostim subcutaneously at 6 μg/kg/day for 56 days. Adverse events for the sargramostim and placebo groups were 100% (10/10) and 80% (8/10), respectively. These included injection site reactions, increased total white cell counts, and upper extremity bone pain. One urticarial and one vasculitis reaction were found to be drug and benzyl alcohol related, respectively. An additional patient with a history of cerebrovascular disease suffered a stroke on study. Unified Parkinson’s disease rating scale, Part III scores in the sargramostim group showed modest improvement after 6 and 8 weeks of treatment when compared with placebo. This paralleled improved magnetoencephalography-recorded cortical motor activities and Treg numbers and function compared with pretreated Parkinson’s disease patients and non-Parkinsonian controls. Peripheral Treg transformation was linked to serum tryptophan metabolites, including L-kynurenine, quinolinic acid, and serotonin. These data offer a potential paradigm shift in modulating immune responses for potential therapeutic gain for Parkinson’s disease. Confirmation of these early study results requires larger numbers of enrolled patients and further clinical investigation.

## Immune modulation: translating the benefits

The immune system modulating drug sargramostim shows promising results in a small clinical trial with Parkinson’s disease (PD) patients. Previous studies have shown that sargramostim increases the number of regulatory T cells, attenuates immune responses, and confers neuroprotection in animal models of neurodegenerative disease. To determine whether these findings translate to humans, Howard E. Gendelman at the University of Nebraska Medical Center, USA, and colleagues examined the effects of sargramostim in 20 patients with PD. Despite the high number of mild to moderate reported adverse events, the drug was generally well tolerated and led to an increase in regulatory T cell number and activity. Moreover, preliminary assessments after 6 and 8 weeks of treatment suggested an overall improvement in the motor skills of patients that received the drug compared with those that received a placebo.

## Introduction

PD, the most common neurodegenerative movement disorder, is a progressive and debilitating disease that affects up to 5 million people worldwide.^1^ Characteristic movement deficits parallel reductions in striatal dopamine and progressive loss of substantia nigra pars compacta dopaminergic neurons and their striatal connections. To date, therapy is nearly exclusively devoted to symptomatic control of disease manifestations.^1^ Considerable research efforts are underway to define disease mechanisms and as such develop novel therapies to affect disease outcomes. Indeed, despite knowledge of disease mechanisms, therapeutic modalities remain palliative.^1^ Considerable evidence supports the notion that immune alterations exist in PD. Such alterations can be modulated for “potential” therapeutic benefit.^2^ Mechanisms that could be harnessed for therapeutic gain rest in transforming neurotoxic innate and adaptive immune responses. Indeed, work performed by a number of laboratories have shown that Lewy bodies, containing aggregated and nitrated α-synuclein (N-α-syn), released into the extraneuronal environment induce activated macrophages and microglia and affect the emergence of effector T cell (Teff) populations.^2–4^ In animal models of PD, brain-infiltrating macrophages and microglia produce pro-inflammatory neurotoxins that damage surrounding nigral neurons. Such neurotoxin-producing cells can exacerbate disease outcomes as mediated by peripheral N-α-syn-induced Teff.^5, 6^ In contrast, regulatory T cells (Treg) maintain immunological tolerance, attenuate inflammation, and can positively modify disease at least in PD animal models.^7, 8^ As neurodestructive Th1 and Th17 cells can be transformed by pharmacological interventions into neuroprotective Treg, a platform has recently come operative to harness immunological responses for therapeutic gain.^3^

Sargramostim (Sanofi US, Bridgewater, NJ) is an Food and Drug Administration-approved human recombinant granulocyte-macrophage colony-stimulating factor known to affect myeloid recovery in patients receiving bone marrow transplantation or cancer therapy along with induction of Treg immune responses.^9, 10^ The latter was shown to protect against nigrostriatal neurodegeneration in rodent PD models.^11, 12^ However, sargramostim administered under regimens to counter myeloablative intervention or as adjunctive neoplastic therapy has mild adverse effects that include increased white blood cell (WBC) counts, injection site reactions, and bone or sternal pain, which may be self-limiting for some within aged populations.^13^ Despite mild adverse effects of sargramostim, we evaluated an immune-mediated neuroprotective compound as a potential therapy for PD. We found that on the whole sargramostim was well-tolerated, and the adverse effects observed were similar to the effects of this treatment in other patient populations. Our results demonstrated that sargramostim produced the expected immune transformation with the emergence of increased numbers and improved function of Treg. These desired immunological endpoints were confirmed by metabolomic and transcriptomic tests, showing engagement of the serum tryptophan metabolites that included L-kynurenine, quinolinic acid, and serotonin. Although the study was not powered for motor activity efficacy, nor was it long-term, positive effects on some patients' motor skill sets were seen. The results are of potential importance and warrant the consideration of larger-scale investigations. The clinical efficacy endpoints examined remain exploratory in nature and are also dependent on larger-scale monitoring.

## Results

### Demographics and baseline hematologic and immune profiles

In all, 22 PD patients and 17 non-Parkinsonian subjects were enrolled and assessed for eligibility. No significant differences in demographics were discernible between the PD patients and 17 non-PD subjects (Table 1). PD patients ranged from 53 to 76 years of age with a median and mean age of 64 with symptoms for 3–14 years (median 6 years and mean 7 years). Compared with non-PD subjects who exhibited a mean age of 65 years of age, the immune and WBC differential profiles for PD patients at entry exhibited increased frequencies of neutrophils and α4β7 integrin-expressing Teffs and Tregs, but decreased levels of CD39+ Tregs and basophils confirmed previously reported immune profiles.^14^

**Table 1:** Demographics and entry study profiles for non-Parkinsonian subjects and PD patients

	Non-Parkinsonian subjects		PD patients	
*Demographics* ^a^	*N*	Mean (SD)	*N*	Mean (SD)
Age (years)	17	65 (7)	20	64 (7)
Time since first symptoms (years)	n/a	n/a	20	7 (3)
Time since diagnosis (years)	n/a	n/a	19	6 (3)
UPDRS III score	n/a	n/a	20	22 (8)

	*N* (Percentage)	*N* (Percentage)
Male sex	9 (53)	16 (80)
Caucasian race	17 (100)	20 (100)
Job with pesticides	1 (5)	2 (10)
Exposure to pesticides	4 (24)	10 (50)
Job with chemical solvents	4 (24)	8 (40)
Job with other chemical fumes	4 (24)	8 (40)
Job with heavy metals	2 (12)	2 (10)
Hematological parameter	Mean (SD)	Mean (SD)
WBC × 10^3^/μL	6.3 (1.4)	6.8 (1.6)
RBC × 10^6^/μL	4.7 (0.4)	4.7 (0.3)
Hemoglobin, g/dL	14.4 (1.1)	14.4 (0.8)
Hematocrit, %	43.2 (6.6)	43.1 (2.2)
Mean corpuscular volume (MCV), fL	91.6 (3.8)	91.3 (4.1)
Mean corpuscular hemoglobin concentration (MCHC), %	33.2 (0.9)	33.4 (0.7)
Red cell distribution width (RDW), %	13.2 (0.7)	13.0 (1.1)
Platelet count × 10^3^/μL	225.6 (37.9)	233.0 (57.6)
Neutrophils, %	61.7 (5.6)	66.0 (6.9)^b^
Lymphocytes, %	25.4 (6.0)	23.2 (5.7)
Monocytes, %	8.5 (1.9)	7.4 (1.3)
Eosinophils, %	2.9 (1.5)	2.5 (1.4)
Basophils, %	1.0 (0.2)	0.7 (0.4)^b^
Neutrophil × 10^3^/μL	3.9 (1.0)	4.6 (1.2)
Lymphocytes × 10^3^/μL	1.6 (0.5)	1.6 (0.5)
Monocytes × 10^3^/μL	0.5 (0.1)	0.5 (0.1)
Eosinophils × 10^3^/μL	0.2 (0.1)	0.2 (0.1)
Basophils × 10^3^/μL	0.1 (0.0)	0.0 (0.0)
T cell panel	Mean (SD)	Mean (SD)
CD3+, %	70.5 (7.1)	71.3 (8.5)
CD3+/μL	1162.7 (456.9)	1123.2 (360.6)
CD4+, %	50.9 (7.2)	49.2 (11.0)
CD4+/μL	835.9 (320.9)	769.4 (263.6)
CD8+, %	18.9 (6.4)	21.3 (8.1)
CD8+/μL	316.3 (183.2)	342.6 (195.6)
CD4+/CD8+ Ratio	3.1 (1.0)	2.9 (1.1)
% Teff/CD4+	1.1 (0.4)	1.1 (0.4)
% α4β7 Integrin+/Teff	8.3 (3.5)	14.5 (10.6)^b^
% Treg/CD4+	5.4 (1.2)	5.4 (1.3)
% FOXP3+/CD4+	8.9 (2.8)	8.7 (2.6)
% CD39+/Treg	55.8 (15.8)	41.6 (23.8)^c^
% α4β7 Integrin+/Treg	6.2 (2.1)	8.6 (3.0)^b^

*n/a* not applicable

^a^ Demographic information obtained from controls and patients at the time of enrollment were used

^b^ *P* < 0.05

^c^ *P* ≤ 0.10 by Mann–Whitney U test

### Sargramostim effects on primary safety endpoints and adverse events

Twenty PD patients randomized to receive sargramostim (*N* = 10) or placebo (*N* = 10) (Supplementary Fig. S1) showed similar demographics and pesticide/heavy metal exposure histories (Table 2). Seventy percent of the PD patients completed the study. All patients treated with sargramostim and 80% of placebo-treated patients reported at least one adverse event; the difference between the sargramostim group (10/10, 100%, 95% CI 72–100) and placebo group (8/10, 80%, 95% CI 49–94) was non-significant (hazard ratio 1.25, 95% CI 0.92–1.70). The most frequently reported adverse events among sargramostim-treated and placebo-treated patients, respectively, were injection site reactions (10/10, 100% vs. 4/10, 40%, *P* = 0.01), abnormal laboratory/WBC counts (10/10, 100% vs. 3/10, 30%, *P* = 0.003) and pain at sites other than injection sites included bone extremities, torso, pain, and chest-tightening (7/10, 70% vs. 3/10, 30%, *P* = 0.179) are all well-known associations with sargramostim administration and were considered mild adverse reactions.^15^ Eosinophil frequencies increased by 8-fold to 16-fold during sargramostim treatment (*P* < 0.0001), and all hematological values returned to baseline by 4 weeks after drug cessation. Physical examination and blood metabolic values were unremarkable during treatment.

**Table 2:** Demographics and adverse events for PD patients

*Demographics*	Placebo		Sargramostim	
	*N*	Mean (SD)	*N*	Mean (SD)
Age (years)	10	67 (6)	10	62 (7)
Time since first symptoms (years)	9	7 (3)	10	7 (2)
Time since diagnosis (years)	10	5 (4)	10	6 (3)
UPDRS III score	10	24 (10)	10	20 (5)

	*N* (Percentage)	*N* (Percentage)
Male sex	8 (80)	8 (80)
Caucasian race	10 (100)	10 (100)
Jobs with pesticides	3 (30)	0 (0)
Exposure to pesticides	7 (70)	3 (30)
Jobs with chemical solvents	4 (40)	4 (40)
Jobs with other chemical fumes	5 (40)	4 (40)
Jobs with heavy metals	1 (10)	1 (10)
Adverse events^a^		
Any adverse event	8 (80)	10 (100)
Any severe adverse events	0 (0)	3 (30)
Any serious adverse events	0 (0)	1 (10)
Adverse event leading to withdrawal	0 (0)	4 (40)
Possible relationship to drug	7 (70)	10 (100)
Definitive relationship to drug	2 (20)	7 (70)
Category^a^		
Injection site reaction	4 (40)	10 (100)^b^
Abnormal laboratory values	3 (30)	10 (100)^b^
Pain, other than injection site	3 (30)	7 (70)
Pain, upper torso and extremities	0 (0)	7 (70)^b,g^
Pain, lower torso and extremities	3 (30)	3 (30)
Chest pain or discomfort	0 (0)	4 (40)
Muscle, soreness, weakness	4 (40)	3 (30)
Rash, other than injection site	2 (20)	4 (40)
Shortness of breath, wheezing	0 (0)	3 (30)
GI tract, nausea, vomiting	0 (0)	3 (30)
Injury	3 (30)	2 (20)
Headache	2 (20)	2 (20)
Fatigue	2 (20)	2 (20)
Infection, any	2 (20)	2 (20)
Neurological, psychological, dyskinesia	2 (20)	2 (20)
Chills, fever	1 (10)	2 (20)
Itching, other than injection site	0 (0)	2 (20)
Cardiovascular, hematological	0 (0)	2 (20)
Skin, not infection	3 (30)	1 (10)
Equilibrium	1 (10)	1 (10)
Sleep anomalies	1 (10)	1 (10)
Edema, other than injection site	0 (0)	1 (10)
Ophthalmological	0 (0)	1 (10)

	Median (IQR)	Mean (SD)	Median (IQR)	Mean (SD)
Severity of combined adverse events^c^	1.2 (1.1–1.4)	1.2 (0.1)	1.7 (1.4–1.8)^e^	1.6 (0.3)
Likelihood of events being drug-related^d^	2.4 (1.9–2.7)	2.2 (0.6)	3.8 (3.1–3.9)^e^	3.6 (0.6)
Severity of injection site reaction^c^	1.5 (1.0–2.0)	1.5 (0.6)	1.0 (1.0–2.0)	1.3 (0.5)
Severity of pain, other than injection site^c,g^	1.0 (1.0–1.8)	1.2 (0.5)	2.0 (2.0–3.0)	2.0 (0.7)
Severity of pain, upper torso and extremities^c,g^	nd^f^	nd	2.0 (1.8–2.0)	2.0 (0.6)
Severity of pain, lower torso and extremities^c,g^	1.0 (1.0–1.8)	1.2 (0.5)	2.0 (1.5–3.0)	2.1 (0.8)

^a^ Adverse events reported since the initiation of placebo/drug. More than two adverse advents per patient may have been reported; however, patients are only counted once within each category. The same patient may be counted in different categories

^b^ *P* ≤ 0.01 by Fisher’s exact test

^c^ Scored by attending physician; 1 = mild, 2 = moderate, 3 = severe

^d^ Scored by attending physician; 1 = Unrelated, 2 = Unlikely, 3 = Possibly, 4 = Probably, 5 = Definite

^e^ *P* ≤ 0.004 by Mann–Whitney U test

^f^ *nd* no data

^g^ The incidence of upper torso bone and musculoskeletal chest pain were higher in patients treated with sargramostim compared with placebo and was distinct from that associated with PD^19–22^

Mean (±standard deviation [SD]) severity scores for all adverse events were greater in the sargramostim group (1.6 ± 0.3) than in placebo group (1.2 ± 0.1) (*P* = 0.004). Event-associated severities for all pain sites between groups were not significantly different and ranged from mild and moderate (Table 2). Frequencies of patients with pain at sites other than injection sites that included upper and lower torso and extremities were not significantly different between treatment groups (*P* = 0.179). Pain severities as scored by the attending physician at injection sites and at sites other than injection sites including the extremities, the lower torso, and the upper torso and included “chest-tightening”, ranged from mild to moderate and were not significantly different between treatment groups (Table 2). Notably, during the 2 months of pre-treatment baseline observations in our study (visits 1–3), three patients reported pre-treatment pain (2/10, 20% in the sargramostim group vs. 1/10, 10% in the placebo group) (data not shown), suggesting that reported pain experienced during treatment was distinguishable from those attributable to PD. However, in the context of staggered enrollment and non-significant event frequencies among treatment groups, adverse events were not considered significant enough to break the study blind. The likelihood of a treatment-associated adverse event was greatest in the sargramostim group (*P* = 0.002) with likelihood scores ranging from possible to probable, while those in the placebo group ranged from unlikely to possible.

Severe events included a generalized hypersensitivity reaction, a leukocytoclastic vasculitis, and a thrombotic stroke; the latter two were deemed unlikely associated with drug. Notably, for the first patient enrolled, sargramostim was formulated with benzyl alcohol as a preservative. Administration of this formulation for 48 days led to the vasculitis, which responded successfully to formulation cessation and steroid treatment with complete symptom resolution. As benzyl alcohol was deemed vasculitis-associated, the preservative was removed from all subsequent preparations without further incidents of vasculitis. The subject who experienced stroke presented a previous history of hypertension and parallel co-morbid vascular events and were confirmed by magnetic resonance imaging examination. As there were no contraindications of sargramostim for cerebrovascular disease the subject remained in and completed the study. The four sargramostim-treated patients who withdrew from study included the two subjects with upper torso, bone pain, or chest-tightening (Supplementary Fig. S1, Table 2). Extensive work up demonstrated that none of these symptoms were linked to cardiac disease that was the precipitating concern that led to drug cessation decisions. Complete data sets for analysis were obtained for 66 visits by sargramostim-treated patients compared with 77 visits for placebo group. Serum anti-sargramostim antibodies were detected in the drug group by week 4 of treatment (visit 5), but diminished by week 8 (visit 7). Antibody levels were marginal at 4 weeks after drug cessation (Supplementary Fig. S2).

### Sargramostim increases CD4+ Treg subsets and Treg-mediated suppression

**Fig. 1:**
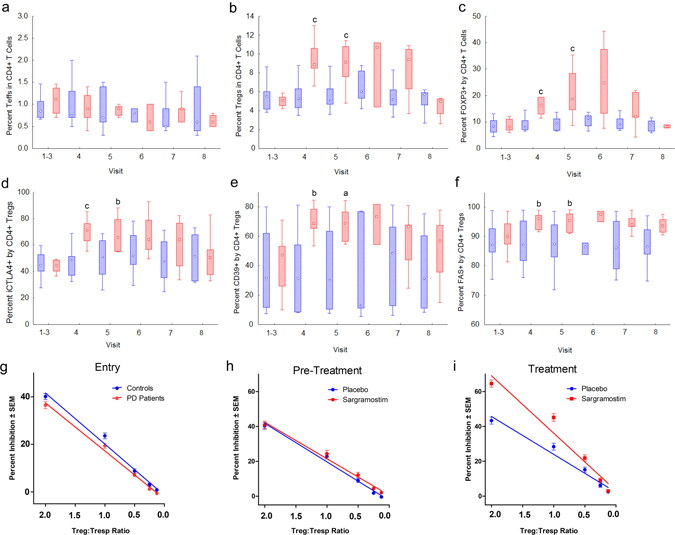
Peripheral blood lymphocytes from PD patients treated with placebo or sargramostim were assessed for the expression of Treg phenotype and function over a 3-month mean baseline (visits 1–3), every 2 weeks after the initiation of treatment (visits 4–7), and 4 weeks after discontinuation of treatment (visit 8). Flow cytometric analyses for percentage of **a** CD4+ Teffs (CD4+CD127hiCD25hi), **b** CD4+ Tregs (CD4+CD127loCD25hi), **c** FOXP3+CD4+ Tregs, **d** iCTLA4+CD4+ Tregs, **e** CD39+CD4+ Tregs, and **f** FAS+CD4+ Tregs. *Plots* represent the medians, interquartile ranges (IQRs) (*boxes*), and non-outlier ranges (*whiskers*) for T cells from PD patients. Levels of T cell subsets from PD patients treated with placebo (*n* = 6–10) (*blue*) or sargramostim (*n* = 5–9) (*red*) were compared by Mann–Whitney U test with *P* ≤ ^a^0.10, ^b^0.05, or ^c^0.01. **g**–**i** Enriched Treg isolates were assessed for the capacity to suppress CD3/CD28-stimulated CD4+CD25− Tresps from a healthy donor. Tregs were serially diluted two-fold and co-stimulated with a constant number of CFSE-stained Tresps to yield decreasing Treg:Tresp ratios. Treg activity as percentage inhibition of proliferation was determined for **g** non-Parkinsonian controls (*n* = 17) and non-allocated PD patients (*n* = 20) at 8, 4, and 0 weeks before treatment initiation (Entry, visits 1–3); **h** for randomized PD patients prior to initiation of treatment (Pre-Treatment, visits 1–3); and **i** at 2, 4, 6, and 8 weeks after initiation (Treatment, visits 4–7). Comparison of differences in slope or elevation as an indicator of Treg activity was determined by linear regression analyses for baseline paired controls and PD patients (*P*_slope_ = 0.49, *P*_elevation_ = 0.065, *n* = 17) (Entry); for baseline of placebo (*n* = 10) or sargramostim (*n* = 10) randomized PD patients (*P*_slope_ = 0.59, *P*_elevation_ = 0.17) (Pre-Treatment); and for PD patients during treatment with sargramostim (*n* = 5–9) compared with placebo (*n* = 9–10) (*P*_slope_ = 0.063, *P*_elevation_ = 0.058) (Treatment). Comparison of Treg activity from pre-treated and treated patients randomized to sargramostim group (*P*_slope_ = 0.039) or placebo group (*P*_slope_ = 0.88, *P*_elevation_ = 0.04)

The study yielded expected Treg induction outcomes. This provides clear evidence that sargramostim treatment can achieve the desired immunological endpoint. Sargramostim treatment resulted in higher numbers of CD3+ and CD4+ T cells (Supplementary Fig. S3b, d) with increasing, though not significant, frequencies when compared with placebo (Supplementary Fig. S3a, c). Frequencies and numbers of CD8+ T cells (Supplementary Fig. S3e, f) and ratios of CD4+ and CD8+ cells (Supplementary Fig. S3g) were unaffected. Frequencies of CD4+ Teffs remained unchanged regardless of whether sargramostim or placebo was administered (Fig. 1a). In contrast, sargramostim treatment increased frequencies of CD4+CD127loCD25hi Tregs as early as 2 weeks, which remained elevated thereafter (Fig. 1b, c). Tregs exhibited higher frequencies of subsets that express CD39 and FAS (CD95), or intracellular CTLA4 (iCTLA4) (Fig. 1d–f). Treg function was assessed as the ability to suppress CD3/CD28-stimulated proliferation of CD4+CD25− Tresps. Baseline Treg function in PD patients was diminished (*P* = 0.07) compared with non-PD subjects (Table 1, Fig. 1g, Entry), thus confirming results from our previous study.^14^ Prior to treatment, Treg function was similar among both randomized PD patient groups (Fig. 1h, Pre-Treatment). In contrast, treatment with sargramostim increased Treg activity compared with pre-treatment (differences in slopes, *P* = 0.04) and to placebo group (differences in slope, *P* = 0.06 and elevation, *P* = 0.07) (Fig. 1i).

### Sargramostim induces immune-linked metabolites and augments suppressor activity

**Fig. 2:**
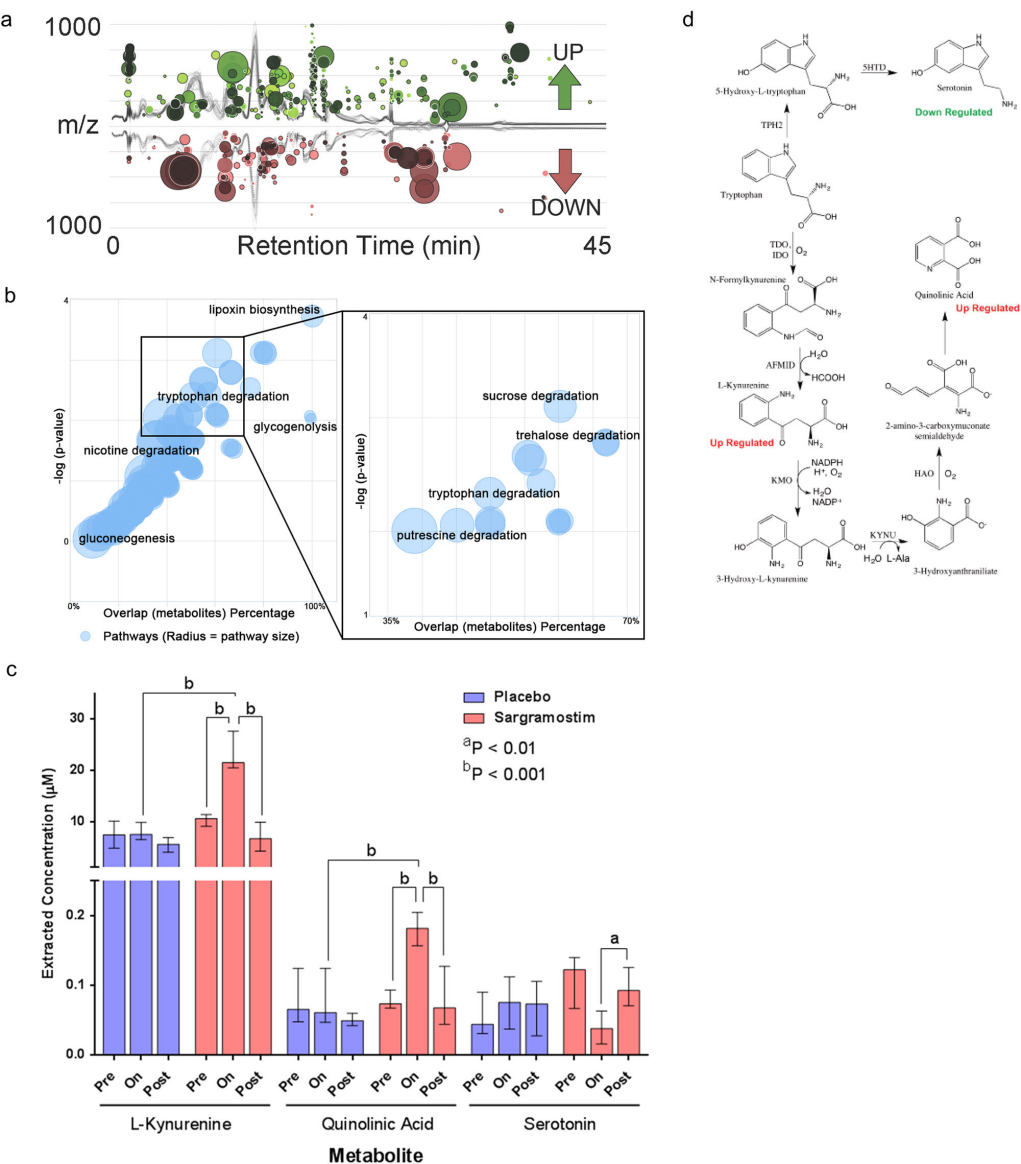
Metabolomic analyses from serum from sargramostim-treated or placebo-treated PD patients. **a** Global metabolomic analysis was performed on serum samples from the entire PD patient cohort. Comparisons of metabolic features were performed between sample groups specifically focusing on pre-treatment groups (visits 1 and 2) vs. on treatment groups (visits 5 and 7), and between treatment groups either on placebo or on sargramostim. A cloud plot illustrating the dysregulated features between pre-treatment and on-treatment are overlaid on the chromatographic runs. Each *circle* represents a dysregulated feature at a specific retention time (*x*-axis) and mass-to-charge ratio (*y*-axis). The diameter of each feature represents the fold change and the color intensity represents the significance (*P*-value). Six-hundred metabolites were found to be upregulated or downregulated in PD patients treated with sargramostim compared with their respective pre-treated controls. **b** The 600 dysregulated metabolites were cross-referenced with known metabolic pathways, analyzed by Welch’s *t*-test to identify dysregulated features, and altered metabolic pathways were determined by using the *mummichog* algorithm which maps possible metabolite matches and targets local enrichments that reflect true pathway activity opposed to false matches that otherwise are randomly distributed.^16^ The plot shows the statistical relevance of dysregulated metabolic pathways for sargramostim-treated patients compared with pre-treated controls as the −log_10_ *P*-value as the function of the weighted mean percentage overlap of metabolite pathway identifying the tryptophan metabolism as a key pathway affected by treatment with sargramostim. The greater color intensity represents a more significant *P*-value and the diameter represents the percent coverage of metabolites found to be dysregulated in a given pathway. **c** Targeted metabolomic analyses of serum from PD patients at pretreatment (Pre, visits 1 and 2), at weeks 4 and 8 during treatment (On, visits 5 and 7), and at 4 weeks after treatment cessation (Post, visit 8). When available, results from the same patient, but at different visits were averaged and binned into pre-treatment or on-treatment. Medians and IQRs of tryptophan metabolite concentrations were determined from patients randomized into placebo group (*blue bars*) (*n*_Pre_ = 8, *n*_On_ = 9, *n*_Post_ = 8) or sargramostim group (*red bars*) (*n*_Pre_ = 9, *n*_On_ = 7, *n*_Post_ = 5). Comparison of median metabolite concentrations between pre-treatment, on-treatment, and post-treatment samples and between samples from placebo-treated and sargramostim-treated groups were determined by Mann–Whitney U tests. Of the 18 targeted metabolites from the tryptophan pathway, many were below the calibration curve or detection limits, or were unchanged. **d** Metabolomic analysis showed concentrations of kynurenine and quinolinic acid upregulated, whereas serotonin was downregulated within the tryptophan pathway. Enzymes in the tryptophan pathway include TPH2, tryptophan hydroxylase-2; 5HTD, 5-hydroxytryptophan decarboxylase; TDO, tryptophan 2,3-dioxygenase; IDO, indoleamine 2,3-dioxygenase; AFMID, arylformamidase; KMO, kynurenine 3-monooxygenase; KYNU, kynureninase; and HAO, 3-hydroxyanthranilate 3,4-dioxygenase

Sargramostim-mediated increases in Treg frequency and function suggested the possible prevalence of systemic conditions conducive for Treg development. To assess that possibility, serum from PD patients prior to, during, and after treatment with sargramostim or placebo were assessed by global untargeted metabolomic analyses. Six-hundred metabolites were dysregulated following sargramostim treatment compared with controls (Fig. 2a). Using the *mummichog* algorithm,^16^ significant alterations in the tryptophan pathway (*P* < 0.002) were found with links to inflammation, immunological tolerance, and Treg function (Fig. 2b). To delineate those alterations, targeted metabolomics for the tryptophan pathway yielded levels of three key metabolites from sargramostim-treated patients that differed significantly from pre-treatment or post-treatment levels and levels from placebo-treated patients (Fig. 2c, d). L-Kynurenine concentration from the sargramostim group was 2.3-fold and 3.0-fold higher than those from pre-treated or placebo-treated patients, respectively; and quinolinic acid concentration was 2.4-fold higher than those from either pre-treated or placebo-treated patients. Both metabolites returned to baseline levels by 4 weeks after treatment. In contrast, serotonin levels from sargramostim-treated patients diminished 2.5-fold (*P* = 0.03) and 2.2-fold (*P* = 0.054) from levels of pre-treated and placebo-treated patients.

### Sargramostim induces a complex pattern of immune activation in CD4+CD25− T cells

**Fig. 3:**
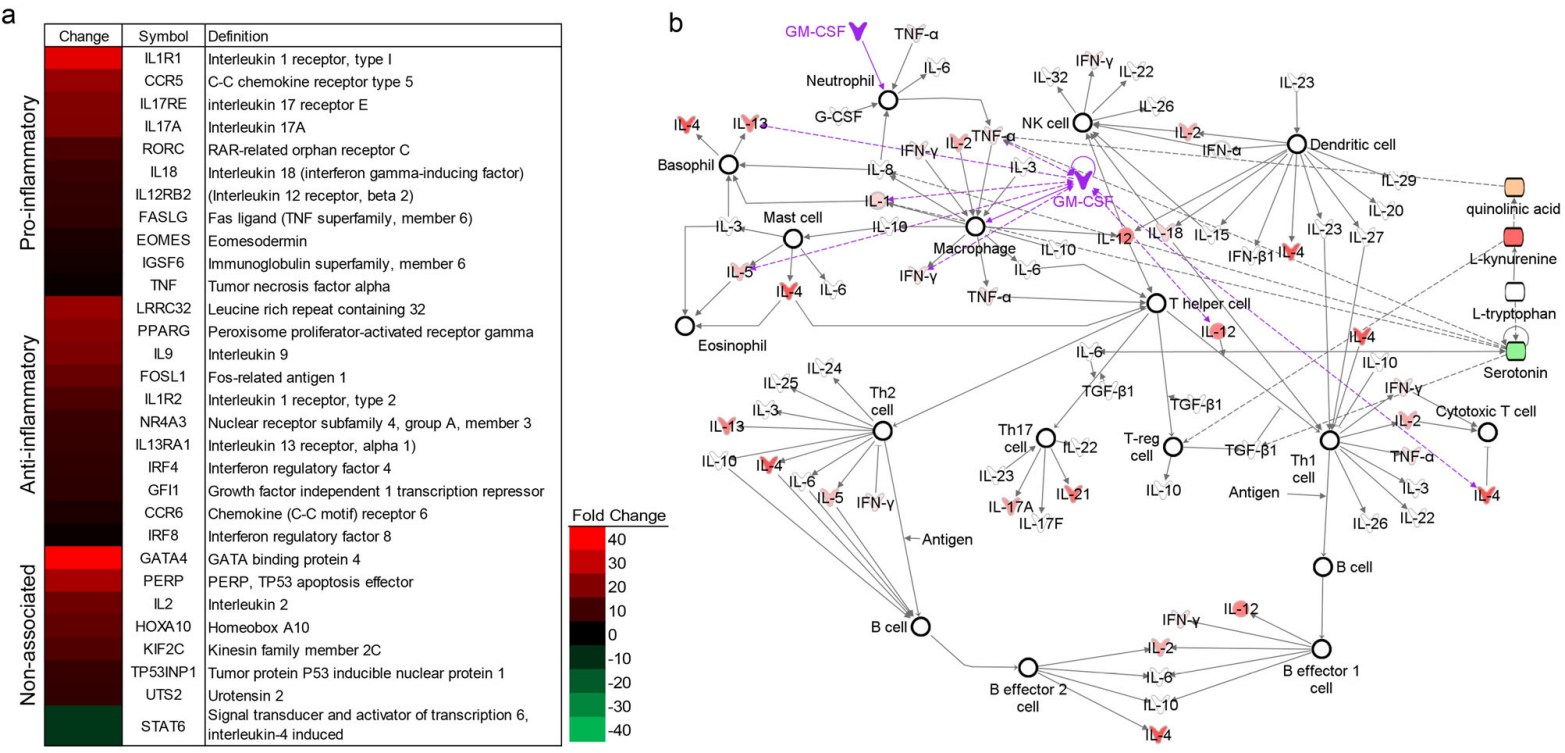
T cell gene expression analyses of T cells from sargramostim-treated or placebo-treated PD patients. **a** Significant increase or decrease in expression of genes by CD4+CD25− T cells from PD patients treated with sargramostim compared with placebo. Genes are divided into those associated with Th1 and Th17 (Pro-inflammatory), Th2 and Tregs (Anti-inflammatory), and general T cell proliferation and differentiation (Non-associated). Significant differences are indicated by a heat map. The map ranged from 40-fold increase (*red*) to 40-fold decrease (*green*). **b** Ingenuity pathway analyses performed on upregulated or downregulated genes to identify putative network associations involved in hematological development and T cell function. Genes and mediators that are upregulated are shaded *red* with the *darker shades* indicating more upregulation; shades of *green* denote downregulation; and nodes in *white* represent putative-associated function

The presence of both pro-inflammatory and anti-inflammatory mediators in sargramostim-treated patients posed putative mechanisms for relationships between immunity and clinical outcomes. Thus, the effects of sargramostim on T cell gene expression were examined in a random subset of patients. Five placebo-treated and four sargramostim-treated PD patients were evaluated. CD4+ T cells were isolated from whole blood and depleted of CD25+ Tregs and Teffs. RNA from CD4+CD25− T cells was isolated and cDNA made for quantitative real-time Polymerase Chain Reaction (PCR) to determine expressed genes linked to Th1, Th2, Th17, and Treg. Expectedly, sargramostim induced a significant upregulation of mRNAs associated with T cell proliferation (*GATA4*, *IL2*, *HOXA10*, and *KIF2C*) (Fig. 3a). Moreover, with increased Treg numbers and function induced by sargramostim, anti-inflammatory *PPARG*, *LRRC32*, *FOSL1*, *IL1R2*, *IL13RA1*, *NR4A3*, and *GFI1* gene expression was increased. Sargramostim upregulated expression of genes associated with pro-inflammatory Th1 and Th17 effectors (*IL17RE*, *IL17A*, *RORC*, *IL18*, and *EOMES*), despite a demonstrated lack of increased Teff numbers in sargramostim-treated patients. These data demonstrate a complex pro-inflammatory and anti-inflammatory gene expression and network interaction by T cells poised for Treg or Teff differentiation during sargramostim therapy (Fig. 3b).

### Sargramostim improves motor and cortical motor activities

**Fig. 4:**
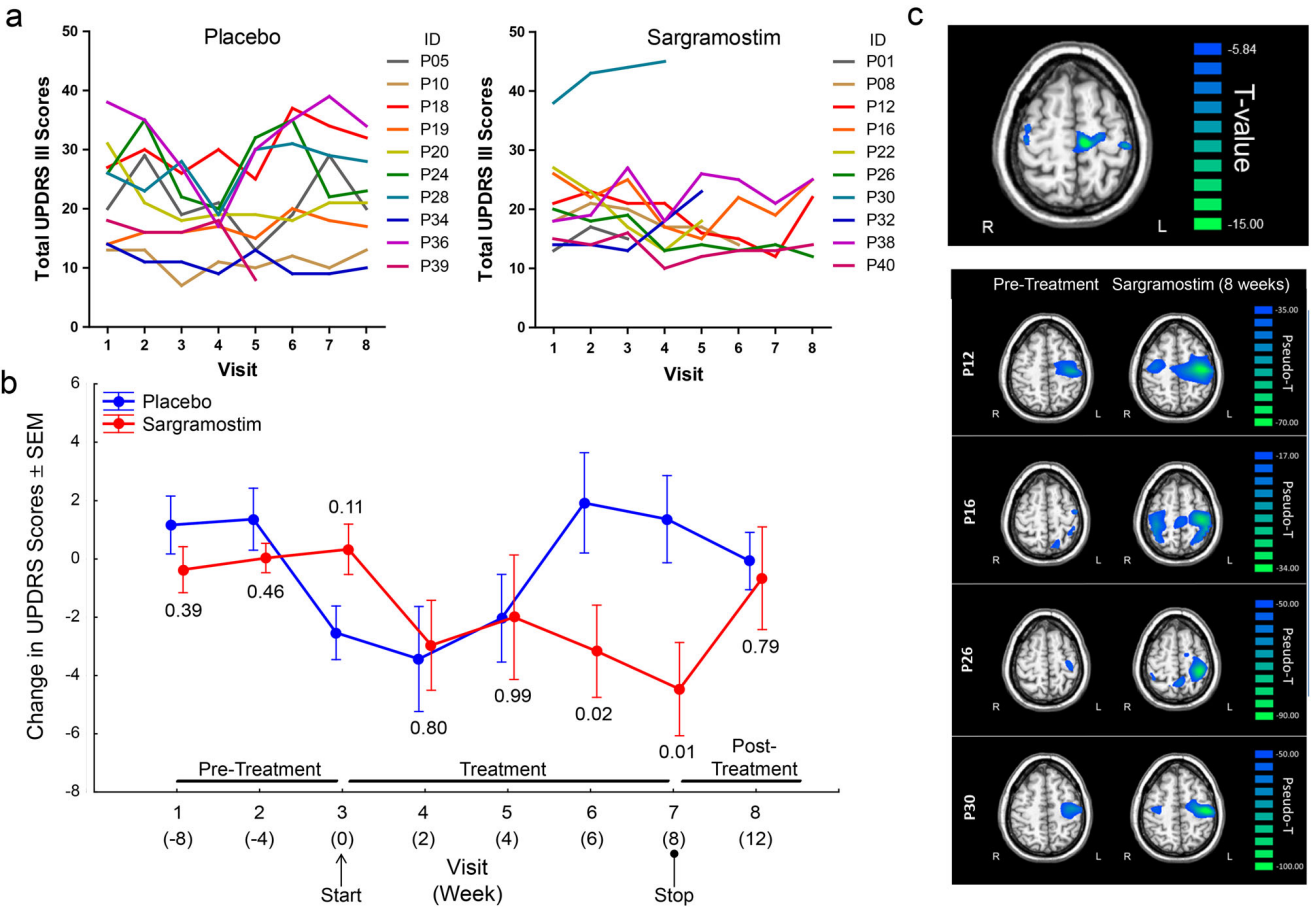
PD patients were randomized to receive placebo or sargramostim. **a** UPDRS III scores of each individual patient were assessed at 0, 4, and 8 weeks (visits 1–3) before treatment (Pre-Treatment); at 2, 4, 6, and 8 weeks (visits 4–7) during treatment (Placebo or Sargramostim); and at 4 weeks (visit 8) after cessation (Placebo or Sargramostim Post-Treatment). Higher scores represent more severe motor symptoms. **b** Changes from baseline UPDRS III scores were determined at each visit for placebo-treated and sargramostim-treated patients using the mean scores of visits 1–3 for each patient as baseline from which to normalize. Changes in scores from each randomized treatment group were normally distributed and homoscedastic by Levene’s test (*P* > 0.05). Factorial ANOVA showed an effect of randomized treatment group (*P* = 0.05) and marginal effects of visit (*P* = 0.07) and treatment-by-time (*P* = 0.05). Fisher’s least significant difference post hoc tests were used to determine pairwise differences between placebo and sargramostim treatment at each visit. UPDRS III scores for placebo group diminished at start of treatment, which may reflect a placebo effect, but returned to baseline during the study course. **c** MEG assessment of beta ERD in PD patients. Paired sample *t*-test comparison of beta ERD activity at baseline (pre-treatment) and during treatment for the group of PD patients receiving sargramostim. Significant increases in beta ERD amplitudes are noted for the pre-treated patient composite in the left and right precentral gyri, right premotor cortex, and SMA (*top panel*). Increases in beta ERD activity from pre-treatment to sargramostim-treatment are shown for individual patients. Compared with pre-treatment, the left precentral gyrus showed a significant effect of visit [*F*(2, 9) = 8.869, *P* = 0.007] and visit-by-group interaction [*F*(2, 9) = 6.04, *P* = 0.022], which was quadratic [*F*(1, 10) = 10.772, *P* = 0.008]. The right precentral gyrus also showed a visit-by-group interaction [*F*(2, 9) = 3.321, *P* = 0.06], which also was quadratic [*F*(1, 10) = 5.447, *P* = 0.04]. The right premotor cortex showed a marginal effect of visit [*F*(2, 9) = 3.050, *P* = 0.07] and the effect was quadratic [*F*(1, 10) = 6.124, *P* = 0.03]. Quadratic interactions were explained as beta ERD amplitudes that increase from pretreatment baseline while on sargramostim and return to baseline levels after termination of treatment

This study was not powered to evaluate clinical outcomes on motor activity, nor was it a long-term study. Thus, the clinical assessments are exploratory in nature. We monitored UPDRS III scores over 2 months (visits 1–3) prior to treatment initiation to establish a pre-treatment baseline. Comparison to baseline, treatment did not appear to worsen motor scores (Fig. 4a). However, while inter-patient variation precluded meaningful statistical analysis of the total UPDRS III scores per visit for each patient, the relative low profiles of scores over visitation from sargramostim-treated patients suggested an overall improvement compared with the placebo group. To confirm that observation, we assessed for each patient, changes from mean baseline score at each visit during pre-treatment (visits 1–3), during treatment (visits 4–7), and after cessation of treatment (visit 8). We showed that compared with placebo treatment, sargramostim showed effects associated with treatment, visit, and treatment-by-visit (Fig. 4b). A transient reduction in score of the placebo group at visit 3 was seen which returned to baseline during the study course. For the sargramostim group, scores diminished over the 8-week treatment period by a mean of 3.1 ± 0.5 (*P* = 0.004) compared with 0.5 ± 1.3 (*P* = 0.78) for the placebo group. The greatest changes in UPDRS III scores were found at 6 and 8 weeks (visits 6 and 7) on sargramostim (Fig. 4b). Score changes returned to baseline by 4 weeks (visit 8) after treatment cessation.

We also used magnetoencephalography (MEG) as a biomarker for motor function. Our previous studies showed decreased beta event-related desynchronization (ERD) amplitudes in the motor hand-knob region of the precentral gyrus in PD patients compared with healthy controls.^17, 18^ In this study, we found no significant differences in beta ERD activity in the placebo group (baseline vs. on-treatment or on-treatment vs. treatment termination) in any motor-related region. In the sargramostim group, beta ERD amplitudes significantly increased from baseline to on-treatment in the left precentral gyrus, right precentral gyrus, right premotor cortex, and supplementary motor area (SMA) (*P* < 0.005, cluster-corrected; Fig. 4c, *top panel*). Notably, each patient exhibited increased beta ERD amplitudes on sargramostim when compared with baseline measures (Fig. 4c, *bottom panel*).

## Discussion

Sargramostim was generally well-tolerated by PD patients. Overall frequencies of patients experiencing adverse events between sargramostim and placebo groups were not significantly different and were confined to increased injection site reactions, increased WBC counts, or upper torso or extremity bone pain; all reported adverse effects to therapy with sargramostim.^13^ Mild side effects such as extremity or torso pain representing bone marrow replenishment are associated with these types of constitutional symptoms such as bone and muscle ache or minor pain and are helped by nonsteroidal drugs or acetaminophen. Sternal or non-cardiac chest pain or “chest tightening” was rated as moderate to severe symptoms. These torso and extremity pains described for sargramostim are readily distinguishable from the common types of pain experience by patients with PD.^19^ Meta-analysis of pain in PD for 18 studies and 15,636 cases revealed a mean prevalence of pain in 58.36% of patients that included a relatively wide variation ranging from 11 to 83%.^20^ The wide variability in prevalence is due, in part, to lack of (1) visibility to the physician, (2) reporting by the patient, (3) treatment priority if reported, and (4) attention to sufficient objective signs that are disregarded.^21^ Distribution of pain types in PD includes musculoskeletal pain (48%), dystonic pain (26%), neuropathic pain (13%), and non-localized/central pain (8%).^20, 22^ Musculoskeletal pain originates from rigidity or skeletal deformity due to Parkinsonism. Dystonic pain is related to involuntary muscular contractions and is often associated with anti-Parkinsonian medication. Neuropathic pain is thought to be related to the central dopaminergic deficit. Non-localized/central neuropathic pain is a burning pain with spontaneous onset and periods of exacerbation. It is poorly localized and is more intense on the affected side.

Sargramostim treatment was associated with significantly transformed immune function and sera metabolites. Treatment-associated improvements in UPDRS III scores and cortical motor electrical activities paralleled one another as well as the immune biomarker changes. These findings are supported by a number of prior animal and clinical studies for PD^2, 3, 5, 11, 14, 23–25^ and other neurodegenerative disorders.^26–28^ Indeed and as in this report, it is now well known that control of pro-inflammatory signals by Tregs is a focus of clinical research activities to develop novel neurotherapeutics for a range of neuroinflammatory and neurodegenerative disorders. By suppression of T cell proliferation and production of a broad range of anti-inflammatory cytokines that includes, for example, interleukin-10 and transforming growth factor-β, Tregs can in fact ameliorate the pathobiology and clinical signs and symptoms of progressive neuronal degeneration. Moreover an imbalance of effector and regulatory immune cells can affect systemic inflammatory and metabolic processes and predict disease progression. This has been previously uncovered for a spectrum of disorders beyond PD that include Alzheimer’s disease, stroke, and amyotrophic lateral sclerosis.^26, 27, 29–31^ Thus, restoring or increasing Treg frequency and enhancing their suppressive activities by immune modulators such as sargramostim is believed to be a novel yet promising approach for treating these disorders.

For PD, N-α-syn, the dominant protein in dopaminergic neuronal inclusions, induces potent neurotoxic Teffs that can accelerate nigrostriatal degeneration.^5, 32^ Transformation of Teff responses by Tregs leads to significant dopaminergic neuronal protection^3^ and proportional changes in numbers of interferon-γ-producing Th1, IL-4-producing Th2, and CD4+CD25+ T cells are linked to the tempo of disease progression.^14, 33^ However, whether sargramostim-induced changes in T cell profiles could affect PD pathobiology remained unknown. This possibility is bolstered by drug correction of PD-associated Treg dysfunction and improved motor task outcomes.

Parallel observations as shown herein were reported in a spectrum of autoimmune and neurodegenerative diseases.^30, 34–37^ Thus for Tregs, defining the tempo, phenotype, and functional role in disease is bolstered by their known abilities to attenuate microglial inflammatory responses and ongoing neurodegeneration. Gene array evaluations showed that sargramostim had multiple effects on peripheral T cells, supporting the idea that an established neuroinflammatory environment was required to affect a regulatory cell profile. This unique idea of cooperative pro-inflammatory and anti-inflammatory neuroprotection was supported by our metabolomics studies. Here, tryptophan pathway dominance was associated with flow cytometric Treg activity. While 5-hydroxytryptophan is converted to serotonin,^38^ tryptophan is, in parallel, converted to kynurenine by indoleamine-pyrrole 2,3-dioxygenase (IDO), and kynurenine is further metabolized to quinolinic acid.^39–41^ IDO expression and kynurenine production induce Treg formation. Notably, IDO can be increased by both anti-inflammatory and pro-inflammatory cytokines^42^ as is seen in Parkinsonian patients.^43^

The clinical observations are exploratory in nature in this study with a relatively small sample size and short duration. It does seem clear, however, that sargramostim does not worsen motor function. The suggestion of improved motor function, along with the apparent biomarker responses measured by MEG, is intriguing and will need to be evaluated more fully in larger studies.

Taken together, our findings show that sargramostim treatment in PD is feasible and reasonably well-tolerated. They support the idea that the effects of sargramostim on T cell polarity change depending on the brain-immune environment. The induction of Tregs, modulation of Teffs, and overall improvement of immune modulatory activities by Tregs is a novel pathway that corrects aberrant immune responses during PD. The therapeutic potential of sargramostim awaits larger confirmatory studies.

## Materials and methods

### Participants and study design

This single-center, randomized, double-blind, phase 1 clinical trial was performed at the University of Nebraska Medical Center (UNMC), Omaha, NE, USA, to test the safety and tolerability of sargramostim for PD. Twenty-two PD patients were recruited from the metropolitan area between 24 September 2013 and 14 August 2015 for an intention to treat design. The final follow-up was 8 January 2016. Inclusion criteria were 35–85 years of age at onset with symptoms of asymmetric bradykinesia and resting tremor or muscle rigidity persisting for ≥3 years, and ≤stage 4 by Hoehn and Yahr disease scale.^44^ Seventeen age-matched non-Parkinsonian subjects served as non-PD controls. Exclusion criteria included multiple system atrophy, corticobasal degeneration, unilateral Parkinsonism lasting of >3 years, prior head injury, stroke, brain surgery, a PD family history of >1 blood relative with the disease, mental illness, cognitive impairment, and autoimmune, systemic inflammatory or hematologic disease. Patients were excluded if administered lithium, neuroleptics, immune modulatory treatment within 90 days of study onset or had allergies to benzyl alcohol, colony-stimulating factors, yeast-derived products, or ferrous metal body implants. The trial was completed as designed.

### Standard protocol approvals, registrations, and patient consents

The research protocol (IRB Protocol 487-12) was approved by the UNMC Institutional Review Board. Patients were identified and referred to the Clinical Research Center (CRC) by their primary physician. Written informed consent was obtained from all participants by CRC personnel following the Good Clinical Practice guidelines. This trial is registered at ClinicalTrials.gov, Identifier: NCT01882010.

### Randomization and masking

PD patients were randomized at a 1:1 ratio to receive sargramostim or placebo. Randomization and assignment was performed at the time of accrual since participant enrollment was staggered. Patients were block randomized by the study statistician in randomly chosen blocks of 2 or 4, and the list was given to the trial pharmacist for drug and placebo preparation. The pharmacist prepared identical syringes of sargramostim and placebo to provide doses necessary for 2 weeks. Examining physicians and medical personnel were blinded to treatment assignment. Randomly generated three-letter codes identified patient blood samples and were used throughout the study to monitor processing, analyses, and safety.

### Procedures

This trial was performed in two parts. In the first part, non-PD subjects and PD patients had three pre-treatment appointments at −8, −4, and 0 weeks (visits 1–3) to determine a comparative baseline; after which, the non-PD subjects were dismissed. In the second part beginning at visit 3, 20 PD patients administered by subcutaneous self-injection either sargramostim at 6 μg/kg/day (10 patients) or a placebo of weight-based volume of saline/day (10 patients) for 56 days.^15^ PD patients continued with appointments every 2 weeks for 2 months (visits 4–7), and a follow-up visit (visit 8) 4 weeks after treatment cessation. All enrolled patients that received at least one treatment dose were analyzed for primary outcomes. Blood samples, physical examinations, and unified Parkinson’s disease rating scale, part III (UPDRS III) evaluations were performed during each visit. The primary neurologist performed UPDRS III assessments in a double-blinded fashion in the “ON” state. All but one patient maintained their individually prescribed anti-Parkinsonian regimen throughout the study.

Study drug was withheld for ~24 h prior to each visit. WBC counts with differentials, immunocyte (leukocyte) numbers, and sera metabolites were monitored. Immunocytes obtained from peripheral blood were stained with fluorochrome-conjugated monoclonal antibodies against CD4 (FITC or AF700), CD127 (PerCP-Cy5.5), CD25 (PE), FOXP3/Scurfin (AF647), CD152/CTLA-4 (APC), CD95/FAS/Apo1 (APC), CD39/ENTPD1 (APC), Integrin β7 (APC) (all BD Biosciences, San Jose, CA) and CD49d/Integrin α4 (PE-Cy7) (BioLegend Inc., San Diego, CA). Isotype-matched antibodies were negative controls. For FOXP3 and iCTLA4, cells were permeabilized with BD Cytofix/Cytoperm kit (BD Biosciences). Cell surface and intracellular T cell epitopes were examined with an LSR II flow cytometer (BD Biosciences). For Treg function, CD4+CD127loCD25hi cells were enriched by negative selection using a Complete Kit for Human CD4+CD127loCD25+ and CD4+CD127lo enrichment (Stemcell Technologies, Vancouver, Canada). CD25+ Tregs were 89 ± 8% (mean ± SD) of the enriched CD4+ cell population. Naïve CD4+CD25− responder T cells (Tresps) were isolated from healthy donors for proliferation tests.^14^ For T cell gene expression, CD4+CD25− T cells were enriched by MACS column negative selection (Miltenyi Biotech, San Diego, CA). mRNA was isolated from Treg-depleted and Teff-depleted CD4+ T cells, reverse transcribed, and cDNA subjected to real-time PCR using the RT^2^ Profiler Human T Helper Cell Differentiation array (Qiagen, Valencia, CA). Fold-changes were determined using the RT^2^ Profiler PCR array data analysis software version 3.5.

Serum was submitted for antibody and metabolomic profiling. IgG or IgM anti-sargramostim antibodies were screened by enzyme-linked immunosorbent assay (ELISA) and immunoprecipitation and titers confirmed by endpoint ELISA and by neutralization tests using a luciferase-reported functional assay. For metabolomics, sera was extracted in acetonitrile/methanol, resuspended in acetonitrile/water, sonicated, and analyzed.^45^ Targeted metabolomic analyses employed reverse phase high-performance liquid chromatograph-mass spectrometry.

Recent reports suggest the utility of MEG in monitoring neurophysiological activity, motor dysfunction, and therapeutic outcomes in PD and other neurodegenerative disorders.^17, 18, 46–49^ For these studies, cortical neurophysiological activity during a right-hand movement task was recorded using high-density MEG.^17, 18^ Activity was recorded at 4 weeks (visit 2) before treatment, 8 weeks (visit 7) after initiation of treatment, and 4 weeks (visit 8) after drug cessation. Participants were recorded after 12 h off Parkinsonian medications. MEG data were individually corrected for head motion and noise.^50^ Artifact-free epochs were transformed into the time-frequency domain, and the movement-related beta ERD response (14–24 Hz, −300 ms to 200 ms, movement onset = 0 ms) was imaged using beamforming.^18, 51, 52^

### Outcomes

The primary study endpoint was safety as monitored by complete blood counts with differentials, blood metabolic panels, adverse events, and UPDRS III scores. Hematologic panels were performed by the hospital’s clinical laboratory. Regimen-blinded neurologists recorded examinations of blood pressure, pulse, skin, lung, liver, heart, and abdomen. UPDRS III scores were measured in the “ON” state. Physical examinations were unremarkable in all cases. Adverse events were recorded in treatment diaries by patients and by physicians who scored (1–3) events by severity as (1) mild, (2) moderate, or (3) severe, and scored (1–5) whether the event was (1) unrelated, (2) unlikely, (3) possible, (4) probable, or (5) definitely related to the study drug. Mild events cause minimal discomfort or concern, may require minimal or no treatment, and do not interfere with daily activities. Moderate events were defined as discomfort, inconvenience, or concerns ameliorated with simple therapeutic measures. Severe adverse events were defined as discomfort or incapacitation that may require prescription drug therapy, other treatments, or interventions. Secondary outcomes were MEG neurophysiological activities, immune phenotype and function, and serum metabolomics.

### Statistical analyses

Sample size estimates of 16 PD and 16 non-PD controls for baseline observations (−8 weeks) were determined to provide 80% power using a two-sided Wilcoxon test assuming normal distribution and a mean percent change from baseline of 0.80. This yielded an overall mean immune response score of 6.32 (SD of 0.97).^14^ To assess sargramostim effects on immune responses, a sample size of eight in each treatment group was determined to provide a 95% CI equal to the sample mean ± 0.81. All participants that received at least one treatment dose were included. Statistical analyses were conducted using SAS/STAT software (version 9.2 or higher; SAS Institute Inc., Cary, NC) or Statistica (version 9, StatSoft, Tulsa, OK), with tests being two-sided. The frequency of adverse events was compared between groups using the Fisher’s exact test (Prism, v6, GraphPad Software, Inc., La Jolla, CA). CD4+ T cell subsets, function, gene expression, antibody titers, and metabolites were compared between treatment groups using a two independent samples *t*-test or Mann–Whitney U test. For MEG, a 2 × 3 mixed-model ANOVA statistical evaluation used peak voxels from each significant brain region with treatment as a between-subjects factor and visit as a repeated factor. For Treg function, percentage inhibition of proliferation was determined at each Treg:Tresp ratio as slope and axis-intercepts by linear regression (Prism, v6). A data and safety monitoring board of UNMC physicians and faculty advised study investigators.

## Electronic supplementary material

Supplementary Fig. S1

Supplementary Fig. S2

Supplementary Fig. S3

## Electronic supplementary material

Supplementary Information accompanies the paper on the *npj Parkinson’s Disease* website (doi:10.1038/s41531-017-0013-5).
