# Supplementary material for: Evaluation of the safety and immunomodulatory effects of sargramostim in a randomized, double-blind phase 1 clinical Parkinson’s disease trial
Source: NPJ Parkinsons Dis. 2017 Mar 23;3:10. doi: 10.1038/s41531-017-0013-5 (PMC5445595; doi:10.1038/s41531-017-0013-5)
Supplement: Supplementary file 1 — Supplementary Fig. S1 [file 41531_2017_13_MOESM1_ESM.pdf]

CONSORT TRANSPARENT REPORTING of TRIALS

CONSORT 2010 Flow Diagram

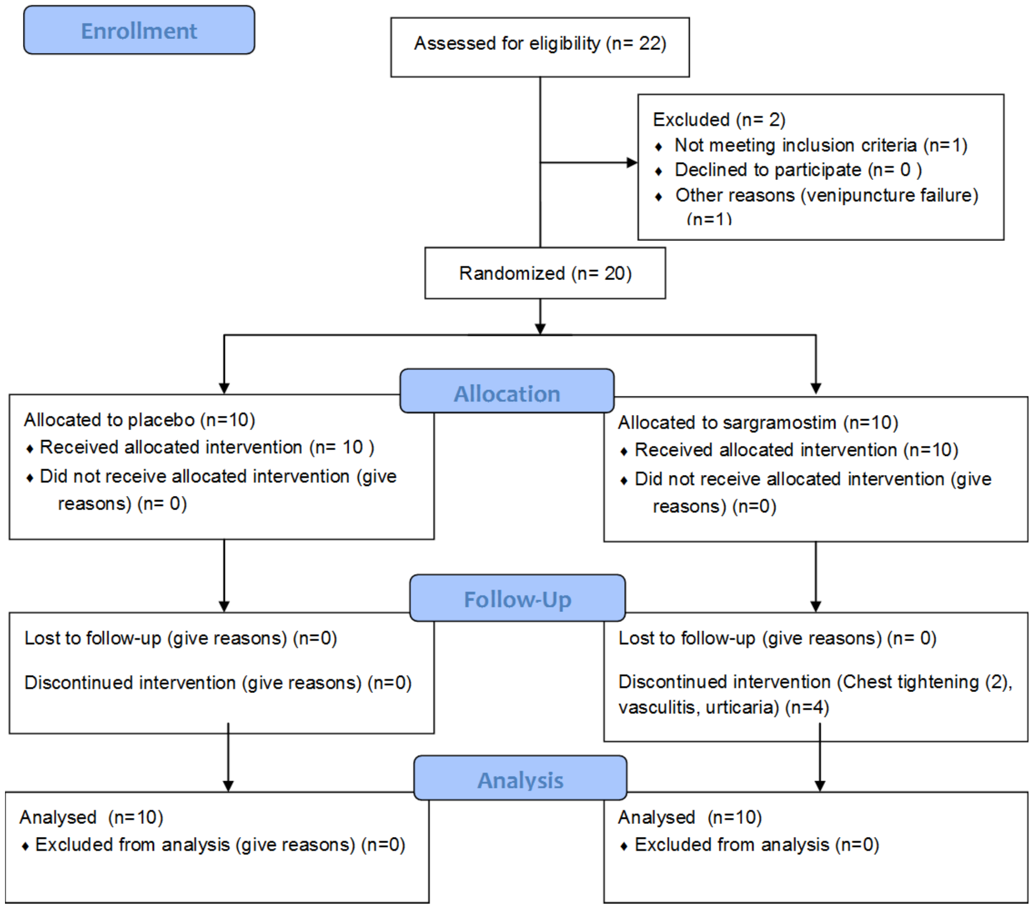

Figure S1. CONSORT flow diagram.

Twenty-two PD patients were initially recruited to participate in an intention-to-treat trial. One patient failed to meet inclusion criteria and another was excluded due to inability to provide blood by venipuncture. Twenty patients were randomized, and 10 patients were allocated to receive sargramostim and 10 to receive placebo. In the sargramostim-treated group, 2 withdrew due to chest-tightness or bone pain, 1 withdrew due to leukoclastic vasculitis, and 1 withdrew due to a urticarial response.
