# Supplementary material for: Evaluation of the safety and immunomodulatory effects of sargramostim in a randomized, double-blind phase 1 clinical Parkinson’s disease trial
Source: NPJ Parkinsons Dis. 2017 Mar 23;3:10. doi: 10.1038/s41531-017-0013-5 (PMC5445595; doi:10.1038/s41531-017-0013-5)
Supplement: Supplementary file 2 — Supplementary Fig. S2 [file 41531_2017_13_MOESM2_ESM.pdf]

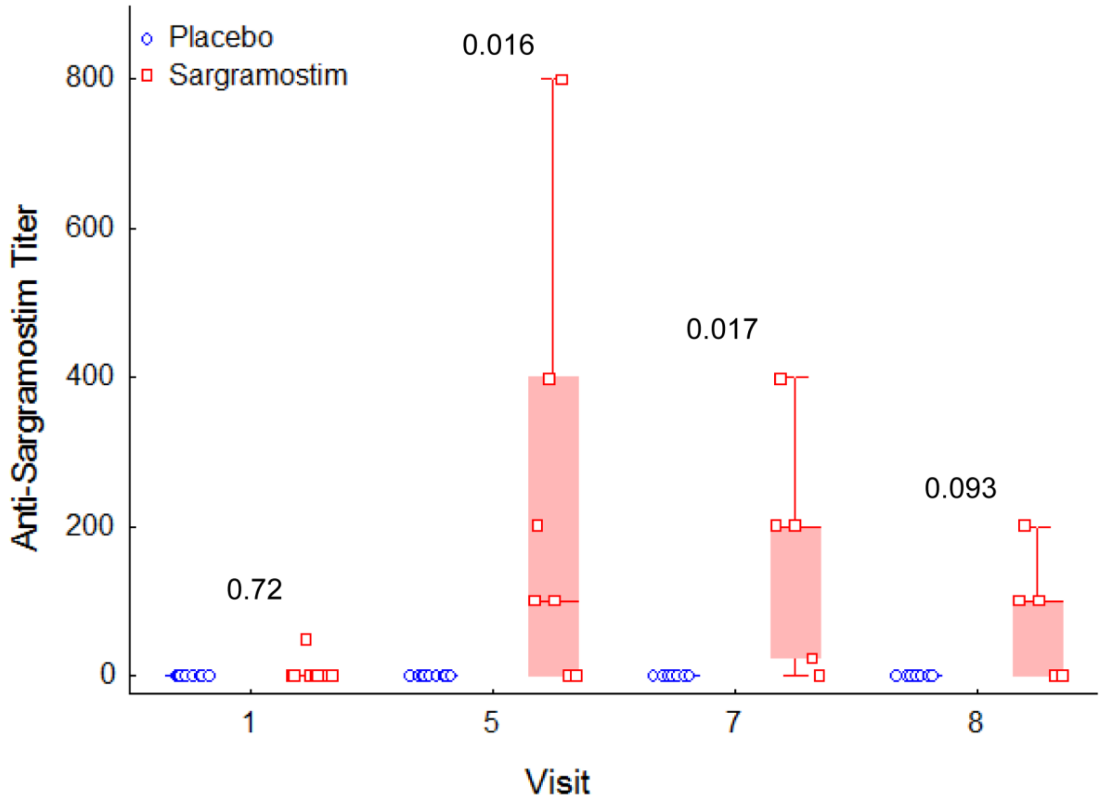

Figure S2. Anti-sargramostim antibodies in sera of treated PD patients.

Serum samples from PD patients treated with placebo (blue open circles) or sargramostim (red open boxes) were obtained at weeks -8, 4, 8, and 12 relative to initiation of treatment (visits 1, 5, 7, and 8). Samples were initially screened by sargramostim-specific ELISA. Positive samples were confirmed by immunoprecipitation assay; ELISA endpoint titers defined as the reciprocal of the last dilution above the titration; and antibody neutralization using a CSF2R-expressing cell-based assay system with a luciferase reporter detecting functional sargramostim. Medians (line), IQRs (box), and non-outlier ranges (whiskers) were determined for ELISA titers of anti-sargramostim antibodies for PD patients treated with placebo (n_{v1} = 8, n_{v5} = 9, n_{v7} = 7, and n_{v8} = 8) or sargramostim (n_{v1} = 8, n_{v5} = 7, n_{v7} = 5, and n_{v8} = 5). Pairwise comparisons of anti-sargramostim titer medians between placebo- and sargramostim-treated patients for each visit were determined by Mann-Whitney U test and p values provided for each visit.
