# Supplementary material for: Evaluation of the safety and immunomodulatory effects of sargramostim in a randomized, double-blind phase 1 clinical Parkinson’s disease trial
Source: NPJ Parkinsons Dis. 2017 Mar 23;3:10. doi: 10.1038/s41531-017-0013-5 (PMC5445595; doi:10.1038/s41531-017-0013-5)
Supplement: Supplementary file 3 — Supplementary Fig. S3 [file 41531_2017_13_MOESM3_ESM.pdf]

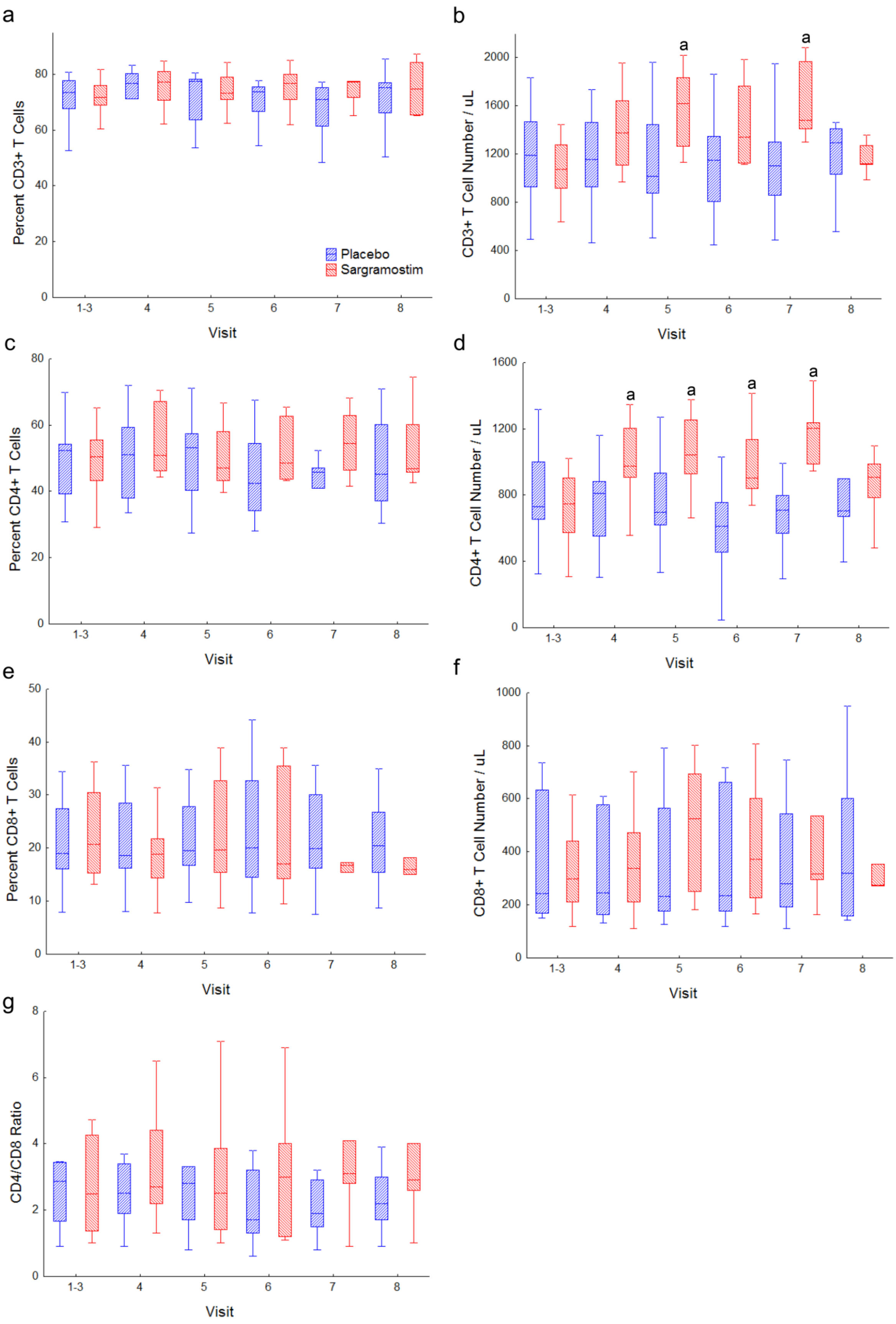
a
Percent CD3+ T Cells
0
20
40
60
80
1-3
4
5
6
7
8
Visit
Placebo
Sargramostim
b
CD3+ T Cell Number / uL
0
400
800
1200
1600
2000
a
a
1-3
4
5
6
7
8
Visit
c
Percent CD4+ T Cells
0
20
40
60
80
1-3
4
5
6
7
8
Visit
d
CD4+ T Cell Number / uL
0
400
800
1200
1600
a
a
a
a
1-3
4
5
6
7
8
Visit
e
Percent CD8+ T Cells
0
10
20
30
40
50
1-3
4
5
6
7
8
Visit
f
CD8+ T Cell Number / uL
0
200
400
600
800
1000
1-3
4
5
6
7
8
Visit
g
CD4/CD8 Ratio
0
2
4
6
8
1-3
4
5
6
7
8
Visit

Figure S3. Flow cytometric analyses of T cells from PD patients.

Peripheral blood lymphocytes from whole blood of PD patients were assessed by flow cytometric analysis for frequency, number, and ratio of T cells (T cell panel) by the UNMC Regional Pathology Services. T cell panels were determined for PD patients over a 3-month mean baseline (visits 1-3), every 2 weeks after the initiation of treatment (visits 4-7), and 4 weeks after discontinuation of treatment (visit 8). Flow cytometric analyses for (**a**) percentages and (**b**) numbers per μL of $CD3^{+}$ T cells; (**c**) percentages and (**d**) numbers per μL of $CD4^{+}$ T cells; (**e**) percentages and (**f**) numbers per μL of $CD8^{+}$ T cells; and (**g**) ratios of percentages of CD+/CD8+ T cells. Plots represent the medians, interquartile ranges (IQRs) (boxes), and non-outlier ranges (whiskers) for T cells from PD patients. Levels of T cell subsets from PD patients treated with placebo (n = 6-10) (blue) or sargramostim (n = 5-9) (red) were compared by Mann-Whitney U test with $P \leq$ [a]0.05.
